# Inhibition Plasticity in Older Adults: Practice and Transfer Effects Using a Multiple Task Approach

**DOI:** 10.1155/2016/9696402

**Published:** 2016-01-14

**Authors:** Andrea J. Wilkinson, Lixia Yang

**Affiliations:** ^1^Department of Psychology, Ryerson University, Toronto, ON, Canada M5B 2K3; ^2^Mechanical and Industrial Engineering, University of Toronto, Toronto, ON, Canada M5S 3G8

## Abstract

*Objective*. To examine plasticity of inhibition, as indexed by practice effects of inhibition tasks and the associated transfer effects, using a multiple task approach in healthy older adults.* Method*. Forty-eight healthy older adults were evenly assigned to either a practice group or a no-contact control group. All participants completed pretest (2.5 hours) and posttest (2 hours) sessions, with a 2-week interval in between. During the 2-week interval, only the practice group completed six 30-minute practice sessions (three sessions per week for two consecutive weeks) of three lab-based inhibition tasks.* Results*. All three inhibition tasks demonstrated significant improvement across practice sessions, suggesting practice-induced plasticity. The benefit, however, only transferred to near-near tasks. The results are inconclusive with regard to the near-far and far-far transfer effects.* Discussion*. This study further extends literature on practice effects of inhibition in older adults by using a multiple task approach. Together with previous work, the current study suggests that older adults are able to improve inhibition performance through practice and transfer the practice gains to tasks that overlap in both target cognitive ability and task structure (i.e., near-near tasks).

## 1. Introduction

### 1.1. Inhibition and Aging

Inhibition is an executive function that keeps cognitive processing (e.g., thoughts and attention) in line with task goals. It is a control process that regulates attention by suppressing to-be-ignored irrelevant items so that attention can be focused on to-be-attended relevant items [1–3]. Inhibition works to control the contents of working memory through* access* (keeping irrelevant information outside one's focus of attention by blocking it from entry) and* deletion* (ridding working memory of no longer relevant information), whereas* restraint* functions to withhold automatic responses that are inappropriate for the task at hand [1, 4]. Deficits in inhibitory processing have been linked to poor performance on tasks of working memory [5], episodic memory [6], and processing speed [7]. These inefficiencies lead to irrelevant information entering one's focus of attention by virtue of a faulty gating mechanism or inefficient removal of no longer relevant information. The result is a short-term memory storage system that is clogged with irrelevant information, which contributes to slower and more inaccurate retrieval [4]. The* inhibitory deficit hypothesis of aging* suggests that many age-related cognitive deficits (e.g., poor memory and slowed processing speed) are the result of poor inhibitory control (e.g., [1]).

In literature, there are several different tasks that measure inhibitory processing, many of which demonstrate age-related declines in performance. For example, using a Local-Global task, Slavin et al. [8] demonstrated a local precedence effect in older adults. The authors showed an attentional preference towards the local (small), as opposed to the global (large) dimension of a stimulus, whereby older adults responded faster to and were more distracted by local relative to global dimension features. Thus, the age-related deficit in the Local-Global task is particularly salient in the local dimension of a stimulus. In addition, seminal work by Kirchner [9] investigated age differences in the* N*-Back task using 0-Back to 3-Back conditions. The results showed no age differences in the 0-Back condition, but incrementally greater age differences with increased *N* in this task (also see [10]). Furthermore, using a modified Go-No Go task, age differences in event-related neural responses to irrelevant “No Go” stimuli have been established [11, 12]. Similarly, the Stroop task (described in more detail below) has also consistently demonstrated reliable age-related decline (e.g., [13–15]).

Inhibition is also very important in everyday life, for example, blocking out surrounding conversations while trying to read the newspaper at a coffee shop or withholding the urge to check e-mails, when trying to write a paper. Furthermore, we all have occasions when it is difficult to concentrate on a train of thought, because recent events or thoughts (pleasant or unpleasant) call our attention too powerfully [6]. Given the critical role of inhibition in older adults' cognition (e.g., memory and speed of processing) and daily lives, the main goal of the current study is to assess the plasticity [16] of inhibition in older adults. Herein, the plasticity of inhibition will specifically be indexed by practice (i.e., improvement in inhibition task performance as a result of practice) and transfer effects (i.e., the degree to which the practice gains can be transferred to other tasks).

### 1.2. Plasticity of Inhibition

#### 1.2.1. Practice Effect

Retest practice effects refer to performance improvement on the target tasks through practice on the same tasks repeatedly across sessions, without any strategy guidance or feedback [17, 18]. Earlier studies have examined the practice effects of inhibition using a single-task approach. For example, the Stroop task has been used to train inhibition in older adults and the results demonstrated improvements within a single session [13], across two sessions [14], or even across six practice sessions [19]. However, little research has explored the plasticity of inhibition using a wider range of inhibition tasks, which will encompass a broader set of inhibitory functions (*access*,* deletion*, and* restraint*) and may involve a wider associated brain network, for the evaluation of potential transfer effects. The current research aims to fill this gap using a six-session multiple task practice approach.

For this purpose, we adopted three practice tasks: Local-Global,* N*-Back, and Go-No Go. These three tasks have a primary focus on the* access*,* deletion*, and* restraint* functions of inhibition, respectively [20–22]. Findings from Wilkinson and Yang [19] suggest that feedback does not moderate the magnitude of the training benefits across sessions; therefore, in the current study, practice was implemented without any adaptive feedback (information on current performance relative to all previous trials). In addition to practice effects, as measured by task performance improvement across sessions, plasticity was also evaluated by the presence of transfer effects (discussed below).

#### 1.2.2. Transfer Effects

Transfer effects refer to the generalizability of the learned skills or performance gains to other tasks or the same task in different contexts [23]. In literature, a hierarchical pattern of transfer effects has been identified, based on the structural and process similarities between the practice and transfer tasks [23, 24]. Following Brainerd's [24] distinction, we intend to assess three levels of transfer effects following inhibition practice: near-near, near-far, and far-far transfer. Herein, near-near transfer refers to improvement in the tasks that measure the same abilities as that being practiced using structurally similar practice tasks, but with varying items, for example, letter* N*-Back (practice task) to digit* N*-Back (transfer task). Near-far transfer refers to improvement in transfer tasks that are different from the practiced tasks, but theoretically tap the same underlying cognitive ability as the practice task, for example, Local-Global, which taps the* access* inhibitory function, as the practice task, and Reading with Distraction as the transfer task (also considered an* access* inhibition task). Last, far-far transfer refers to improvements in tasks that are structurally different from the practiced tasks and tap different cognitive abilities than those being practiced [25]. For example, tasks measuring general cognitive functions that are not specific to inhibition will be considered as far-far transfer tasks.

Previous work indicates that transfer is most likely to occur when the practice and transfer tasks share common underlying processes [26, 27]. In literature, near-near transfer (same ability, similar task) has been successfully demonstrated in older adults following training/practice of basic fluid intellectual abilities [23, 28] and executive functions such as dual-task processing (e.g., [29]), task-switching [25], and inhibition using spatial* N*-Back practice [30]. However, near-far transfer (same ability, different task) is harder to elicit than near-near transfer and is typically shown only in young adults. For example, practice on a letter memory updating task showed transfer to the* N*-Back task in young, but not older, adults [26, 31].

Finally, far-far transfer is rarely elicited in older adults (e.g., [30, 31]), but it is possible. For example, far transfer has been demonstrated in older adults following executive function task-switching practice to other tasks measuring inhibition, spatial working memory, and reasoning [25]. The authors theorized that the far transfer that they found (even in older adults) was due to the various executive processes that were trained using a task-switching paradigm (e.g., goal maintenance, task-set selection, and ignoring irrelevant information), which shared underlying cognitive features with the far transfer tasks. Therefore, practicing on multiple tasks might be a promising approach to maximize breadth of the transfer effects.

Given the above, the current study adopted a multiple task inhibition practice approach to address the following two research questions: (a) Do older adults demonstrate practice effects in all three inhibition tasks: Local-Global,* N*-Back, and Go-No Go? (b) Does practice in the three different inhibition tasks elicit broad transfer to near-near, near-far, and/or far-far transfer tasks in older adults? This approach allows us to pinpoint which task-related features (ability overlap, task structure similarity, or both) are critical to elicit transfer effects in older adults following inhibition practice. It was hypothesized that all three inhibition tasks would show improvement with practice and elicit near-near transfer effects. Furthermore, despite the previous contradictory findings, transfer to far-far tasks has been demonstrated in older adults (e.g., [25]) and may be demonstrated following a multiple task inhibition practice approach.

## 2. Method

### 2.1. Participants

Forty-eight older adults (34 females, age range = 60–88 years;* M* age = 70.25,* SD* = 7.79) were recruited to participate in this study. They were evenly and randomly assigned to either a practice or a no-contact control group. The practice and control group did not differ in any baseline cognitive performance or demographic variables (all *p* values > .25; see Table 1).

All participants provided informed consent according to the Research Ethics Board of Ryerson University. Three participants were suspected for possible colour blindness, as indicated by difficulty in answering five items on the Dvorine Pseudo Isochromatic Plates [32]. Follow-up analyses on Stroop task performance revealed that their data did not affect the findings, so they were included in the final results. All participants had reasonable to normal near vision, with correction if applicable (range 20/20–20/50), as measured with the Rosenbaum near-acuity pocket vision screener [33]. No participants showed dementia-related cognitive impairment; all scored below the cut-off score of six on the Short Blessed Test [34]. No participants reported severe anxiety, as reflected in scores (<26) on the Beck Anxiety Inventory [35]. All participants were debriefed and compensated $10/hour.

### 2.2. Design and Procedure

Participants completed a 2.5-hour pretest session, followed by two weeks of a practice manipulation (i.e., practice versus control), and then completed a 2-hour posttest session. During the 2-week interval, the practice group was instructed to complete six 30-minute lab-based practice sessions (3/week), whereas the control group did not receive any task-related instructions.

### 2.3. Materials and Stimuli

A 17-inch monitor PC was used for all the computerized tasks. Participants were comfortably seated in a well-lit testing room at a viewing distance of approximately 60 cm from the monitor.

#### 2.3.1. Practice Materials and Stimuli

At each practice session, participants completed three tasks: Local-Global,* N*-Back, and Go-No Go, with the order of the tasks counterbalanced across participants. To minimize item-specific effect, the specific letter stimuli used were varied across sessions.

*Local-Global*. The Local-Global task was modeled after Kotchoubey et al. [36], Navon [37], and Thomas et al. [38]. Participants were instructed to attend to either the large (global) or the small (local) dimension of the stimulus (letter) and respond with one of two target letter options (e.g., “A” or “D”). There were three different types of trials. In congruent trials, the to-be-attended and to-be-ignored dimensions were matched (e.g., a large letter A composed of small letter As). In incongruent trials, the two stimulus dimensions were mismatched and both were target letters (e.g., a large letter A composed of small letter Ds). In neutral trials, the two stimulus dimensions were also mismatched, but the to-be-ignored dimension was a control stimulus (e.g., a large letter A composed of small letter Hs for global dimension focus or a large letter H composed of small letter Ds for local dimension focus).

Participants completed two blocks, one in local and one in global dimension focus, counterbalanced across participants. Each block started with 12 practice trials followed by 72 experimental trials. Each trial began with a fixation-cross presented at the centre of the screen for 500 ms, which was replaced by a single stimulus (forced response). Feedback (on accuracy and reaction time [RT]) was provided during practice, but not during the experimental blocks. Responses were made by using the left or right index finger to press the “z” or “{/}” keys labeled with the target letters (e.g., “A” and “D”). The key assignment to targets was counterbalanced across participants. Following the seminal work of Navon [37], the dependent variable was the RT interference score (local and global) calculated by subtracting the mean RT of congruent trials from incongruent trials (i.e., RT_incongruent_ − RT_congruent_).

*N-Back.* The* N*-Back task was modeled after Braver et al. [39]. There were three experimental blocks: 1-Back, 2-Back, and 3-Back, presented in ascending order, each containing 9 target trials and 36 nontarget trials. Stimuli were selected from 20 consonant letters presented in upper and lower case (excluding vowels and “Y”). Participants were instructed to report whether the current letter stimulus was the same as the one presented immediately before (1-Back condition), the 2nd-item back (2-Back), or the 3rd-item back (3-Back) in the series. Three practice blocks—one for each condition—of 10 trials each were provided prior to the experimental blocks.

Each block started with an alerting cue (*∗∗∗∗∗*) presented for 1000 ms followed by a blank screen for 500 ms. Each trial started with a letter stimulus presented at the centre of the screen for 500 ms, which was replaced by a centrally presented fixation-cross for 2000 ms. Participants were instructed to respond during the presentation of the fixation-cross. Basic performance feedback (i.e., “Correct!,” “Incorrect,” or “No response detected”) was presented after each response for 1500 ms, before proceeding to the next trial. Participants did not, however, receive any adaptive feedback that informed performance on the current trial relative to all previous trials. Key assignment (“z” or “/” for “TARGET” or “NONTARGET”) was counterbalanced across participants but kept consistent within participants across sessions. Following Verhaeghen and Basak [10], accuracy measures the likelihood that an item is available for processing and is susceptible to item decay and/or interference from previously presented items. Thus, the dependent variable was overall accuracy for each condition. As *N* increases, the amount of interference within working memory also increases.

*Go-No Go.* The Go-No Go task was modeled after Wilkinson and Yang ([19]; also see [40–42]). In this task, participants were instructed to press the space bar when a single prespecified “Go” stimulus (e.g., “O”) appeared and to withhold their response when a prespecified “No-Go” stimulus (e.g., “X”) appeared on the screen. One block of 30 practice trials (20 “Go” and 10 “No Go” trials) was followed by 200 experimental trials (150 “Go” and 50 “No Go” trials).

Each trial began with a fixation-cross presented at the centre of the screen for 1000 ms, followed by a “Go” or “No Go” stimulus presented centrally for 500 ms or terminated by a key press. Following Wilkinson and Yang [19], as well as Falkenstein et al. [42], the dependent variable was the false alarm rate (i.e., pressing the space bar on a “No Go” trial), which was calculated by dividing the number of committed false alarms by the total number of “No Go” trials.

#### 2.3.2. Pretest and Posttest Materials and Stimuli

A battery of cognitive tasks was administered at pretest and posttest sessions to assess three levels of transfer effects: near-near, near-far, and far-far transfer (see Table 2). To minimize item-specific effects, we used parallel versions of the transfer tasks at pretest and posttest sessions.

*Near-Near Transfer.* Local-Global,* N*-Back, and Go-No Go tasks with varying items (i.e., digits instead of letters) were administered as the near-near transfer tasks at the pretest and posttest sessions. Specifically, letters were used as stimuli in the practice tasks, whereas digits were used as stimuli in the corresponding transfer tasks. The transfer tasks were structured following the same trial procedure as the practice tasks. For the digit Local-Global task, digits 1, 2, 3, and 4 (with target stimuli as 1 and 4 or 2 and 3) were used at pretest and 5, 6, 7, and 8 (with target stimuli as 5 and 6 or 7 and 8) at posttest. The digit* N*-Back task used numbers ranging from 1 to 9. In addition to the conditions practiced during the practice sessions, a 0-Back block was included, whereby participants had to indicate whether a prespecified number (e.g., “5”) appeared at all. In the digit Go-No Go task, number pairs of 1 and 9 or 4 and 8 were utilized, counterbalanced across pretest and posttest sessions. Within each pair, the number assignment to the “Go” and “No Go” condition was counterbalanced across participants. Instead of 150 “Go” and 50 “No Go” trials, one trial list (out of 4) had 152 “Go” trials and 48 “No Go” trials. In this case, the proportion of false alarms was calculated by dividing the number of false alarms by 48 instead of 50. 

*Near-Far Transfer.* The near-far transfer tasks included Stroop, Reading with Distraction, and Directed Forgetting. These tasks are different from the tasks used during practice, but all have been documented to assess inhibition [19, 43, 44].

*Stroop*. The Stroop task was modeled after Wilkinson and Yang ([19], adapted from [45]). The task included three types of trials: congruent (e.g., the word “BLUE” printed in blue ink, respond blue), incongruent (e.g., the word “BLUE” printed in green ink, respond green), and neutral (e.g., “XXXX” printed in blue ink, respond blue). Participants completed three blocks in the following sequence: (1) the key-colour acquisition block (40 trials) aimed at familiarizing participants with the mapping between response keys and the corresponding ink colors; (2) the practice block (24 trials) was the same as the experimental block, but participants received feedback (on accuracy and RT) after each trial to practice the task rules; and (3) the experimental block (216 trials) was the same as the practice block except no feedback was given following each trial. Following our previous work [46], the dependent variable was the Stroop RT ratio interference score that was calculated by dividing the RT of incongruent trials by that of neutral trials (RT_incongruent_/RT_neutral_). 

*Reading with Distraction*. This task was modeled after Connelly et al. [43]. Participants were instructed to read the italicized words of a short passage out loud and ignore the distracting materials that appeared in the display. There were two types of passages: low distracting (ignore string of Xs) and high distracting (ignore words that were not italicized albeit relevant to the passage). Participants then answered four 6-option multiple-choice questions about the passage. For high distracting passages only, one of the multiple-choice response options was a to-be-ignored word. Four different passages (two high distracting and two low distracting) were presented at each session. There were two dependent variables: a reading speed difference score (RT_high_ − RT_low_) and the proportion of multiple-choice distractor intrusions. 

*Directed Forgetting*. This task was modeled after Sego et al. ([47]; also see [48]) and included three blocks: encoding, filler task, and recognition. During encoding, participants saw 24 individually presented words (12 to-be-remembered [TBR] and 12 to-be-forgotten [TBF] words), followed by a cue to either REMEMBER or FORGET the word for a later memory test. Next, participants were asked to judge 50 completed math equations (e.g., 2 + 3 = 5) for accuracy. This filler task was used to reduce selective rehearsal of the TBR or TBF items. Last, during recognition, participants were surprisingly asked to recognize all of the words presented during encoding and indicate if they were OLD or NEW. Thirty-six words, all 24 words from the encoding phase plus 12 new words, were presented. The dependent variable was the hit rate (i.e., proportion of “old” responses to “OLD” words) for TBR and TBF words.

Similar to the practice Local-Global task, Stroop task performance was indexed with an interference score. Thus, the Stroop task could be considered as a near-far “same dependent variable” transfer task. In contrast, Reading with Distraction and Directed Forgetting assess inhibition at a more conceptual level by examining reading speed (RT), intrusion rates, and long-term memory performance (i.e., hits). Therefore, these tasks could be considered as near-far “different dependent variable” transfer tasks. 

*Far-Far Transfer.* The far-far transfer tasks assessed working memory, episodic memory, reasoning, and processing speed with Corsi Block, Word List Recall, Letter Series, and Digit Symbol, respectively.

*Corsi Block*. A computerized version of the Corsi Block visuospatial working memory span task ([49], modified from [50]) was used to assess working memory. The item set size at each trial ranged from 4 to 7, presented in ascending order. In this task, participants were presented with a display of nine grey squares on a white background for 1200 ms. Next, some of the squares would turn black—for 1000 ms each—one at a time in a sequence. Participants were asked to remember and then reproduce the sequence of squares that turned black by clicking the mouse cursor on the squares. Participants first completed six practice trials (three of each 2-span and 3-span), followed by 12 experimental trials (three of each 4-span, 5-span, 6-span, and 7-span). The dependent variable was overall accuracy (i.e., the proportion of trials correctly recalled in the right sequence). 

*Word List Recall*. Episodic memory was assessed with a Word List Recall test [51]. Participants were given three minutes to study the word list. Then, following two filler tasks, Letter Series and Digit Symbol (described below), they were asked to write down all of the words they could recall in the right sequence. The dependent variable was the proportion of correct responses recalled in the correct sequence.

*Letter Series*. The Letter Series task [52] was used to assess inductive reasoning. In this test, participants are tasked with filling in the blank and responding with the next letter that would continue the pattern (e.g., for the series “z  f  y  e  x  d  …,” the correct response would be “w”). The number of correct responses was used as the dependent variable.

*Digit Symbol*. Processing speed was assessed with the Digit Symbol task [53]. In this task, participants were given two minutes to draw corresponding symbols for a series of digits based on a digit symbol conversion code. The number of correct responses was used as the dependent variable.

For the Word Recall, Letter Series, and Digit Symbol tasks, two parallel versions were adopted from our previous work [18, 51] and counterbalanced across participants and pretest versus posttest sessions.

## 3. Results

### 3.1. Statistical Analyses

The data were analyzed using IBM SPSS Statistics 22. To assess practice effects, three repeated measures ANOVAs were conducted: 2 (dimension: local versus global) × 6 (session) ANOVA on RT interference scores for the Local-Global task; 3 (condition: 1-Back, 2-Back, and 3-Back) × 6 (session) ANOVA on overall accuracy for the* N*-Back task; and 6 (session) ANOVA on the false alarm rate for the Go-No Go task. To best capture the nature and trajectory of the practice benefits, all session effects were specified in linear (suggesting incremental improvement) and quadratic contrasts.

To assess transfer effects, mixed model ANOVAs involving session (pretest versus posttest) and group (practice versus control) were conducted for each dependent variable of the transfer tasks. The transfer effect was indexed by a significant 2 (session: pretest versus posttest) × 2 (group: practice versus control) interaction. For Local-Global, 2 (dimension: local versus global) × 2 (session) × 2 (group) ANOVA was conducted on RT interference scores; for* N*-Back, 4 (condition: 0-Back, 1-Back, 2-Back, and 3-Back) × 2 (session) × 2 (group) ANOVA was conducted on overall accuracy; for Go-No Go, 2 (session) × 2 (group) ANOVA was conducted on the false alarm rate; for Reading with Distraction, two 2 (session) × 2 (group) ANOVAs were conducted on passage reading time (difference score) and multiple-choice performance (i.e., distractor intrusions); for Directed Forgetting, 2 (word type: TBR versus TBF) × 2 (session) × 2 (group) ANOVA was conducted on the hit rate; for Stroop, 2 (session) × 2 (group) ANOVA was conducted on Stroop ratio interference scores; for Corsi Block, 2 (session) × 2 (group) ANOVA was conducted on accuracy; for Word List Recall, 2 (session) × 2 (group) ANOVA was conducted on proportion of correct response; and, for Letter Series and Digit Symbol, two 2 (session) × 2 (group) ANOVAs were conducted on the number of correct responses.

### 3.2. Practice Effects

#### 3.2.1. Local-Global

**Figure 1:**
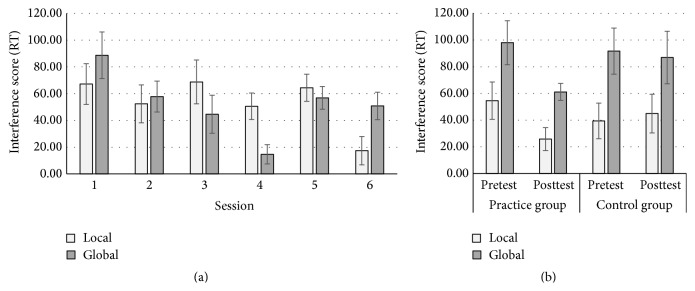
Practice effect (a) and transfer effect (b) of the Local-Global Task. Practice effect refers to the performance improvement (i.e., reduced RT interference scores) across six practice sessions. Transfer effect was indexed by the performance improvement from pretest to posttest in the practice group, but not the control group. Error bars represent the standard error.

Results revealed a significant linear session effect, *F*(1,23) = 6.13, *p* = .02, and *η*_*p*_^2^ = .21, suggesting incremental reduction in interference scores across sessions. The main effect of dimension was not significant, *F* < 1, and *p* = .89. There was also a significant session × dimension interaction in the quadratic contrast, *F*(1,23) = 12.56, *p* = .002, and *η*_*p*_^2^ = .35, but not in linear contrast, *p* = .92 (see Figure 1). In order to tease apart this interaction, separate repeated measures (session) ANOVAs were run for the local and global dimensions. For the local dimension, there was a significant linear session effect, *F*(1,23) = 7.08, *p* = .01, and *η*_*p*_^2^ = .24. Visual inspection suggested that this effect might be primarily driven by session 6. In support of this speculation, the session effect was not significant (*p* = .80), when the analysis was repeated excluding session 6. For the global dimension, the session effect was significant in quadratic contrast, *F*(1,23) = 20.96, *p* < .001, and *η*_*p*_^2^ = .48 (linear, *p* = .06), suggesting that this interaction is driven by a “U” shaped reduction in interference from the local dimension in the global focus condition paired with relatively unchanged interference from the global dimension in the local focus condition. This result suggests practice-induced benefits, indexed by the reduction in the well-reported age-associated local precedence effect, in older adults.

#### 3.2.2. *N*-Back

**Figure 2:**
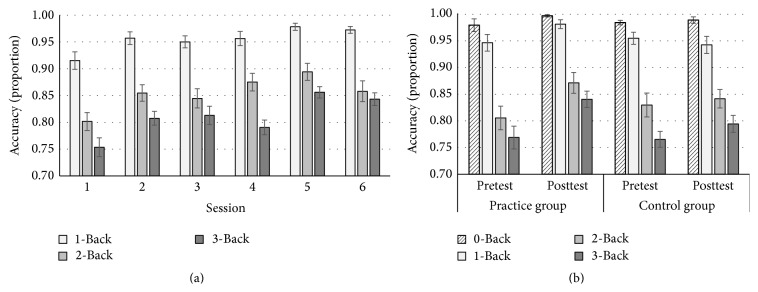
Practice effect (a) and transfer effect (b) of the* N*-Back task. Practice effect refers to performance improvement (i.e., increased accuracy) across six practice sessions. Transfer effect was indexed by the performance improvement from pretest to posttest in the practice group, but not the control group. Error bars represent the standard error.

There was a main effect of condition, *F*(2,46) = 97.78, *p* < .001, and *η*_*p*_^2^ = .81. Follow-up comparisons demonstrated that accuracy significantly reduced from 1-Back (.96) to 2-Back (.86) and then to 3-Back (.81), all *p*  values < .001. Importantly, there were significant linear, *F*(1,23) = 39.56, *p* < .001, and *η*_*p*_^2^ = .63, and quadratic session contrasts, *F*(1,23) = 8.14, *p* = .01, and *η*_*p*_^2^ = .26 (see Figure 2). However, the session × condition interaction was not significant in either contrast, all *p*  values > .14. This suggests an equivalent practice benefit across all three task conditions.

#### 3.2.3. Go-No Go

**Figure 3:**
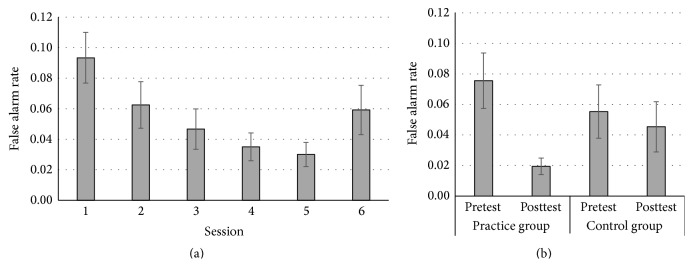
Practice effect (a) and transfer effect (b) of the Go-No Go task. Practice effect refers to performance improvement (i.e., reduced false alarm rate) across six practice sessions. Transfer effect was indexed by the performance improvement from pretest to posttest in the practice group, but not the control group. Error bars represent the standard error.

There were significant linear, *F*(1,23) = 13.20, *p* = .001, and *η*_*p*_^2^ = .37, and quadratic session contrasts, *F*(1,23) = 20.94, *p* < .001, and *η*_*p*_^2^ = .48, suggesting a reduction in false alarms rates with practice (see Figure 3).

All three inhibition tasks demonstrated significant practice effects, suggesting plasticity of inhibition in older adults across all tasks.

### 3.3. Transfer Effects

#### 3.3.1. Near-Near Transfer

*Local-Global*. There was a significant session × group interaction, *F*(1,45) = 4.16, *p* = .05, and *η*_*p*_^2^ = .09 (see Figure 1). Follow-up pairwise comparisons revealed that while the practice group demonstrated significant reductions in interference from pretest to posttest (*M*s = 80.95 versus 43.49, resp.), *p* = .01, the control group showed no change (*M*s = 65.57 versus 65.95, resp.), *p* = .97. The high order 3-way dimension × session × group interaction was not significant, *p* = .67. 

*N-Back*. There was a significant session × group interaction, *F*(1,44) = 8.49, *p* = .01, and *η*_*p*_^2^ = .16 (see Figure 2). Follow-up pairwise comparisons showed that while the practice group performed more accurately at posttest relative to pretest (*M*s = .92 versus .88, resp.), *p* < .001, the control group showed no change (*M*s = .89 versus .88, resp.), *p* = .41. The high order 3-way condition × session × group interaction was not significant, *p* = .41. 

*Go-No Go*. There was a significant session × group interaction, *F*(1,46) = 8.21, *p* = .01, and *η*_*p*_^2^ = .15 (see Figure 3). Pairwise comparisons revealed that only the practice group showed a reduced false alarm rate at posttest relative to pretest (*M* = .02 versus .08, resp.), *p* < .001. The control group showed no change (*M*s = .05 versus .06, resp.), *p* = .39.

#### 3.3.2. Near-Far Transfer

*Reading with Distraction and Directed Forgetting*. Both of these tasks were considered as near-far transfer tasks. None of the dependent variables revealed a session × group interaction (all *p* values > .20 and *η*_*p*_^2^s < .035; see Table 3). 

*Stroop*. The Stroop task was considered as a near-far same dependent variable transfer task. The analysis on the Stroop ratio interference scores revealed that the critical session × group interaction was not significant, *F*(1,46) = 1.80, *p* = .19, and *η*_*p*_^2^ = .04 (see Table 3). The main effect of group was not significant, *F* < 1, and *p* = .603; however, visual inspection and follow-up analyses suggested that the Stroop ratio interference scores were significantly reduced from pretest to posttest in the practice group (*M*s = 1.21 versus 1.15, resp.), *p* = .01, but not for the control group (*M*s = 1.18 versus 1.16, resp.), *p* = .29. Due to a lack of significant interaction, however, this finding must be interpreted with caution.

#### 3.3.3. Far-Far Transfer

None of the dependent variables for the far-far transfer tasks (Corsi Block, Word List Recall, Letter Series, or Digit Symbol) revealed a significant session × group interaction (*p* values > .09 and *η*_*p*_^2^s < .06; see Table 3).

These results provide evidence for robust near-near transfer effects following the practice of three inhibition tasks. Despite some cautionary evidence for near-far transfer to the Stroop task, no strong evidence was detected for near-far or far-far transfer effects.

## 4. Discussion

Inhibition is important in everyday life, for example, keeping our attention focused on the road while driving, even though our grandchildren are screaming in the backseat for ice cream. Given the critical role of inhibition in older adults' cognition (e.g., memory and speed of processing) and activities of daily living, this study aimed to assess the plasticity of inhibition among older adults with a multiple task approach by evaluating (a) the effect of practice on three inhibition tasks, Local-Global,* N*-Back, and Go-No Go, and (b) the associated transfer effects (near-near, near-far, and far-far).

### 4.1. Practice Effects

The practice benefits were evaluated in terms of linear and/or quadratic contrasts of the performance improvement across practice sessions. Linear contrasts typically suggest incremental learning as a result of practice. We speculate that a significant quadratic contrast might indicate saturation or temporary stability/fluctuation of performance at the later practice sessions, probably due to fatigue or lowered effort. Both linear and/or quadratic contrasts were found significant for all three inhibition tasks, demonstrating plasticity of inhibition among older adults (i.e., practice-induced performance improvement).

#### 4.1.1. Local-Global

In line with previous research demonstrating the local precedence effect (i.e., attentional preference and thus larger interference from the local dimension of a stimulus during global dimension focus) in older adults [8], the benefits of practice appear to be more pronounced when the interference comes from the local, rather than the global, dimension in the current study. These findings suggest that practice with the Local-Global task can effectively diminish the local precedence effect, an effect commonly seen in older adults (e.g., [8]). Overall, this indicates that practice enables older adults to be more effective at regulating their attentional focus to reduce interference from the salient to-be-ignored local dimension.

#### 4.1.2. *N*-Back

As expected, performance accuracy on the* N*-Back task gradually decreased as the task demands on inhibition increased (i.e., accuracy of 1-Back > 2-Back > 3-Back), an effect that is exacerbated with age [9, 10]. It should be noted that, in addition to inhibition, the* N*-Back task has a strong working memory component, because participants have to hold target information (i.e., letters or numbers) in their focus of attention, for a certain period of time, to enable them to respond accordingly. As the number of items to be held in working memory increases, working memory load increases, and performance is more vulnerable to interference [10]. Although* N*-Back is a common working memory task, it does require constant updating and deletion of previously, but no longer, relevant information from working memory. In this way, inhibition is an important component of working memory performance. In the current study, all task conditions showed significant and equivalent practice effects, which suggests that, with practice, older adults become more efficient at both keeping relevant information in working memory and removing/deleting no longer relevant information from their focus of attention. By reducing interference from previously presented items (via deletion/inhibition), one makes more room to store and process relevant information in working memory.

#### 4.1.3. Go-No Go

The false alarm data analysis showed a reduced false alarm rate in the Go-No Go task across practice sessions. In line with our previous work [19], this suggests that older adults are able to improve their ability to withhold an automatic motoric response that is inappropriate for the task at hand.

### 4.2. Transfer Effects

#### 4.2.1. Near-Near Transfer

After practicing inhibition using multiple tasks, the results of the current study showed clear and robust near-near transfer effects across all three tasks: Local-Global,* N*-Back, and Go-No Go. Of note, the* N*-Back near-near transfer task may have been rendered more difficult than the practice task due to a reduced stimulus set size. In particular, only 9 digits were used in the* N*-Back transfer tasks, compared to 20 consonant letters used in the* N*-Back practice task. The smaller stimulus set of the transfer task was likely to heighten the possibility of proactive interference (i.e., when previously learned information interferes with current processing) due to enhanced familiarity with the stimuli (e.g., [54]). Despite the possibility of enhanced difficulty of the transfer* N*-Back task relative to the practice* N*-Back task, the near-near transfer effects remained evident. This highlights the robust nature of the near-near transfer effects, which were established following a multiple task approach to inhibition practice among older adults.

In line with previous work (e.g., [29]), the near-near transfer findings demonstrate that older adults maintain the capacity to transfer trained skills to tasks that are structurally similar but with varying items. This suggests that structural and process-based similarities—in combination—between the practice and transfer inhibition tasks are critical for eliciting transfer effects among healthy older adults. In addition, since we varied the items used in the practice (letters) and transfer tasks (digits), the results are also consistent with Yang et al. [18] in that they suggest that inhibition practice benefits are not item-specific. We speculate that the practice and near-near transfer effects demonstrated herein may be primarily driven by increased testing sophistication (i.e., mastering effective strategies and skill learning) and familiarity with the task procedure and structure across sessions.

#### 4.2.2. Near-Far Transfer

For the Stroop task, the results suggested a trending near-far transfer effect. Only the practice group, but not the control group, showed improved performance at posttest relative to pretest. However, we should interpret this finding with caution, given the overall session by group interaction failed to reach significance. No significant transfer effects were revealed for the Reading with Distraction or Directed Forgetting tasks.

One possible explanation for the promising, but limited near-far transfer to the Stroop task is the similarity in the dependent variable between the Stroop transfer task and the Local-Global practice task (i.e., interference score). In contrast, the dependent variables used for Reading with Distraction (i.e., reading speed and distractor intrusions) and Directed Forgetting (i.e., hit rate) did not overlap with those used in any practice tasks, possibly explaining the lack of transfer effects therein. It is possible that the different dependent variables of similar tasks may reflect different aspects of the same ability and thus may vary the magnitude of the transfer effect.

In addition to this, some other important factors should be discussed. For example, differences in task structure, task requirements, and the type and quality of stimuli may also explain the inconsistency or absence of near-far transfer effects. Specifically, for the Reading with Distraction and Directed Forgetting tasks, word stimuli were used and the tasks required more semantic processing of words, such as reading comprehension and/or memory. In contrast, all three tasks used as practice or near-near transfer tasks (i.e., Local-Global,* N*-Back, and Go-No Go) used individually presented digit or letter stimuli and required faster responses largely based on perceptual processing of the stimuli. In this way, differences in basic task stimulus features (words versus digits/letters) and task processing demands (semantic reading/memorization versus perceptual identification/matching) between the practice and transfer tasks may account for the lack of near-far transfer effects to these two tasks.

#### 4.2.3. Far-Far Transfer

Regarding the far-far transfer effects, the study is inconclusive. This is in line with previous research [26, 31]. However, this finding also contradicts a recent meta-analysis on executive-control and working memory practice in older adults [3]. But we note that findings from this meta-analysis have recently been challenged by a reanalysis of these data [55].

In light of the inconsistency in literature, the far transfer effect following executive function training is far from clear. This calls for additional consideration of the design of the practice program. For example, previous studies that have successfully demonstrated far transfer in older adults following cognitive practice have implemented programs that continuously adapt task difficulty based on participants' individual level of performance (e.g., [56]). Therefore, practice or training programs that are continually challenging may keep up participants' engagement levels and thus be more likely to promote far transfer. In line with this discussion, a comparison between training procedures (adaptive, randomized, and self-selected levels of task difficulty) in working memory training among young adults has been evaluated in a recent publication [57]. They found a significant improvement in working memory performance across sessions and no near-far or far-far transfer effects in all groups. In other words, the different approaches to modifying task difficulty within the training program did not modulate training or transfer effects in young adults. However, a gap remains regarding how the training procedure would affect working memory training and transfer effects with increasing age. Future research should explore this research question using an older adult sample, as it is also important to consider that far transfer is difficult to elicit in the aging brain (see [26]).

## 5. Limitations

Although this study makes substantial contributions to the literature, it also has some limitations. First, we utilized a no-contact control group. Previous work [55] suggests that this is the weakest form of control, as it is unclear whether the observed near-near transfer effects have to do with the practiced tasks, setting (e.g., being challenged in a new environment), and/or experience with the investigator. Of note, passive no-contact control groups—where participants are not contacted or provided any instruction for a prespecified amount of time—have been commonly used in training protocols to assess transfer (e.g., [30, 31, 58]). The alternative is an active control group in which participants are guided to participate in other activities that are purposefully engaging the individual (e.g., a physical training program or a series of educational lectures [59, 60]). Of note, an active control may impact transfer because participants are engaged, and the overall engagement level may affect performance on the transfer tasks [56]. Following this logic, it is possible that the near-near transfer effects revealed in the current study might have been overestimated due to the use of a no-contact control group [55]. Future research would benefit from evaluating the differential benefits of a multiple task approach to practicing inhibition using an active versus no-contact control group. For example, an active control group could complete the same number of practice sessions using similar tasks, but without the inhibition requirement, for example, only using congruent and neutral trials in the Local-Global task and the 0-Back condition of the* N*-Back task.

Second, the inconclusive findings regarding near-far and far-far transfer may have been due to the design of the study and/or the sample size of the two groups. For this study, we adopted a six-session practice design based on the literature on age-related cognitive training (e.g., five sessions in [29], four sessions in [25], and six sessions in [19]). However, the small number of practice sessions may have limited the transfer effects. Indeed, far-far transfer has been demonstrated in older adults with a more extensive 20–25 days' training schedule (e.g., [56]); of note, Brehmer et al. [56] also used an adaptive training approach in which task difficulty was adapted to the participants' performance level across sessions. Nevertheless, our results demonstrated that far transfer effects (near-far and far-far) were limited or absent in older adults following six sessions of multiple task inhibition practice. Regarding the sample size, a post hoc power analysis was run using G^*∗*^Power 3.1.9.2 software [61] for repeated measures ANOVA to detect a significant within-between interaction. This analysis revealed sufficient statistical power, of .83 and .80, given the sample size and design, to detect medium (Cohen's *d* = .5) and small effect sizes (i.e., *d* = .2), respectively [62, 63].

The last limitation that warrants mention is the lack of neuroimaging data. This restricts our ability to interpret the limited near-far and absence of far-far transfer effects in terms of the amount of overlap in brain activation patterns between the practice and transfer tasks (see [26]). Future research should explore the overlap in brain activation patterns between a multiple versus single task approach to inhibition practice and the associated hierarchical transfer effects (near-near, near-far, and far-far) in healthy older adults.

## 6. Implications and Conclusions

Given that the ultimate goal of cognitive practice and training programs is to generalize gains beyond the specific practice tasks, the limited near-far and lack of far-far transfer in the current study and literature (e.g., [26, 31]) urge researchers to investigate new approaches to train cognitive abilities in older adults. Current empirical findings suggest that the ideal practice/training programs should focus more on the use of innovative approaches to target changes in thinking patterns using real-world materials (e.g., [64, 65]) rather than on changing specific, discrete cognitive abilities using lab-based computerized tasks. Furthermore, incorporating practice/training tasks that adapt difficulty level to individual performance may also facilitate transfer (see [56]).

In sum, the current study demonstrates that all three inhibition tasks show sizable practice-induced plasticity in older adults (i.e., practice effects). However, the benefits following practice on multiple inhibition tasks was shown to only be transferred to tasks that share both cognitive ability and task structure with the practice tasks (i.e., near-near). The transfer effects to other inhibition tasks (i.e., near-far) or tasks measuring other untrained abilities (i.e., far-far) are limited or nonexistent.

## Figures and Tables

**Table 1:** Demographic characteristics and baseline cognitive performance assessed at pretest separately for the practice group and control group.

Characteristic	Practice group (*n* = 24)	Control group (*n* = 24)	* p*	*d*
Age (years)	68.96 (8.13)	71.54 (7.37)	.26	.33
Gender (female : male)	17 : 7	17 : 7	—	—
Education (years)	15.83 (3.56)	15.71 (2.97)	.90	.04
Health	8.43 (1.34)^*δ*^	8.58 (1.25)	.70	.12
Visual acuity	26.25 (6.47)	27.29 (5.89)	.56	.17
Shipley Vocabulary Test	36.63 (1.97)	35.96 (2.79)	.34	.28
Beck's Anxiety Inventory	4.63 (4.03)	6.17 (6.78)	.34	.28
CES-D	8.83 (6.16)^*δ*^	9.92 (8.52)	.62	.15
Short Blessed Test	.75 (1.29)	.71 (1.52)	.92	.03

*Note.* Standard deviations are in parentheses. CES-D = Centre for Epidemiological Studies of Depression Scale. Education was indexed by the average number of years of formal education. Health was indexed by a self-reported score out of 10. Visual acuity was indexed by the near-visual acuity score from the Rosenbaum Visual Acuity Pocket Screener (score 20/—). Shipley Vocabulary Test was scored by the average number of correct solutions. Average scores were displayed for Beck's Anxiety Inventory, CES-D, and Short Blessed Test.

^*δ*^ *n* = 23; *p* value from the independent *t*-test (practice versus control); *d* = Cohen's *d* effect size calculated for between-subjects comparison.

**Table 2:** List of tasks administered at pretest, practice, and posttest sessions.

Task	Pretest	Practice	Posttest
Local-Global^a^	✓	✓	✓
N-Back^a^	✓	✓	✓
Go-No Go^a^	✓	✓	✓
Reading with Distraction^b^	✓		✓
Directed Forgetting^b^	✓		✓
Stroop^b^	✓		✓
Corsi Block^c^	✓		✓
Word List Recall^c^	✓		✓
Letter Series^c^	✓		✓
Digit Symbol^c^	✓		✓

*Note*. ^a^Near-near transfer task, ^b^near-far transfer task, and ^c^far-far transfer task.

**Table 3:** Performance on the near-far and far-far transfer tasks.

Task	Practice group (*n* = 24)		Control group (*n* = 24)		*p*	*η*_*p*_^2^
	Pretest	Posttest	Pretest	Posttest		
Reading with Distraction^b^ (reading speed difference score)	28.80 (20.91)	27.03 (24.51)	25.23 (19.76)	24.85 (24.39)	.69	.003
Reading with Distraction^b^ (distractor intrusions)	.21 (.09)	.22 (.13)	.21 (.12)	.26 (.15)	.47	.011
Directed Forgetting^b^ (TBR/TBF)	.89 (.17)/75 (.20)	.86 (.16)/.77 (.20)	.86 (.15)/.75 (.20)	.94 (.10)/.77 (.20)	.21	.034
Stroop^b^	1.21 (.12)	1.15 (.09)	1.18 (.09)	1.16 (.09)	.19	.038
Corsi Block^c^	.61 (.14)	.63 (.13)	.60 (.17)	.62 (.14)	.78	.002
Word List Recall^c^	.40 (.35)	.47 (.37)	.45 (.31)	.44 (.31)	.38	.017
Letter Series^c^	10.50 (4.28)	10.83 (4.68)	10.37 (4.17)	12.08 (4.06)	.10	.059
Digit Symbol^c^	61.46 (15.37)	65.42 (15.65)	62.25 (14.79)	65.46 (17.77)	.68	.004

*Note*. Mean scores with standard deviations presented in parentheses. TBR = to-be-remembered; TBF = to-be-forgotten. ^b^Near-far transfer task. ^c^Far-far transfer task. Reading with Distraction was assessed by passage reading speed (difference score) in seconds, and multiple-choice question performance (proportion of distractor intrusions); Directed Forgetting was evaluated by the hit rate for TBR/TBF words; Stroop was measured by Stroop ratio interference scores; Corsi Block was indexed by accuracy (proportion correct); Word List Recall was indexed by accuracy (proportion correct); Letter Series and Digit Symbol were indexed by accuracy (number correct).

*p* values index transfer effects, referring to the session (pretest versus posttest) × group (practice versus control) interaction of the mixed model ANOVA on each dependent variable of the transfer tasks.
